# circKLF4 Upregulates *Klf4* and *Endoglin* to Promote Odontoblastic Differentiation of Mouse Dental Papilla Cells *via* Sponging miRNA-1895 and miRNA-5046

**DOI:** 10.3389/fphys.2021.760223

**Published:** 2022-02-09

**Authors:** Yue Zhang, Hao Zhang, Guohua Yuan, Guobin Yang

**Affiliations:** The State Key Laboratory Breeding Base of Basic Science of Stomatology and Key Laboratory of Oral Biomedicine of Ministry of Education, School and Hospital of Stomatology, Wuhan University, Wuhan, China

**Keywords:** circRNA, KLF4, dental papilla cells, odontoblasts, differentiation

## Abstract

circular RNAs (circRNAs) is a broad and diverse endogenous subfamily of non-coding RNAs, regulating the gene expression by acting as a microRNA (miRNA) sponge. However, the biological functions of circRNAs in odontoblast differentiation remain largely unknown. Our preliminary study identified an unknown mouse circRNA by circRNA sequencing generated from mouse dental papilla and we termed it circKLF4. In this study, quantitative real-time PCR and *in situ* hybridization were used and demonstrated that circKLF4 was upregulated during odontoblastic differentiation. Gene knockdown and overexpression assays indicated that circKLF4 promoted odontoblastic differentiation of mouse dental papilla cells (mDPCs). Mechanistically, we found that circKLF4 increased the linear KLF4 expression in a microRNA-dependent manner. By mutating the binding sites of microRNA and circKLF4, we further confirmed that circKLF4 acted as sponge of miRNA-1895 and miRNA-5046 to promote the expression of KLF4. We then also found that ENDOGLIN was also up-regulated by circKLF4 by transfection of circKLF4 overexpression plasmids with or without microRNA inhibitor. In conclusion, circKLF4 increases the expression of KLF4 and ENDOGLIN to promote odontoblastic differentiation *via* sponging miRNA-1895 and miRNA-5046.

## Introduction

Neural crest derived dental papilla cells play pivotal role in odontoblast differentiation, during which multiple signaling molecules, receptors, and transcription factors have been implicated in mediating odontoblast differentiation (Zhang et al., 2005). Odontoblasts are differentiated cells that produce dentin and (Dassule et al., 2000) and dental papilla cells are usually used to investigate odontoblast differentiation mechanism *in vitro* (Thesleff et al., 1987). With the in-depth research on the odontoblast differentiation, the molecular research mechanism about non-coding RNAs (ncRNAs) was implicated in this differentiation processes (Sun et al., 2015).

ncRNAs play crucial roles in many biological processes. As a member of ncRNAs family, circular RNAs (circRNAs) have become new research hotspots with the rapid development of high-throughput sequencing (RNA-seq) and bioinformatics recently (Sun et al., 2020). Unlike the linear RNAs with 5′ and 3′ termini, circRNAs is a new class of RNA composed of covalently single-stranded closed circular structure due to alternative splicing and back-splicing processes (Memczak et al., 2013; Ashwal-Fluss et al., 2014). Lacking 5′-caps and 3′-tails render circRNAs higher degrees of sequence conservation and stability (Capel et al., 1993; Pasman et al., 1996). circRNAs are ubiquitous across multiple species and the abundance of the circRNAs is approximately 5–10% of their linear counterparts for circular RNA isoforms of most genes. Furthermore, it is estimated that the circular transcript isoforms of some genes are more abundant than linear isoforms for some genes (Salzman et al., 2012, 2013). Besides, circRNAs exhibits tissue-specific and development stage-dependent patterns, which indicates its important role in regulating physiological activities (Memczak et al., 2013; Salzman et al., 2013; Szabo et al., 2015; Greene et al., 2017). Emerging evidence has revealed that circRNAs participate in osteogenesis of periodontal ligament stem cell (PDLSCs) by interacting with miRNA (Gu et al., 2017). Moreover, 154 differentially expressed circRNAs were found to associate with osteogenic differentiation in MC3T3-E1 cells (Qian et al., 2017). In our preliminary study, using circRNA sequencing, we detected the differential expression of circRNAs in mouse dental papilla cells (mDPCs) cultured in either growth medium or differentiation medium for 9 days. Based on these data, we found that 3,255 and 809 circRNAs were upregulated and downregulated after 9 days’ induction of differentiation. These circRNAs profiles suggested that the differentially expressed circRNAs had specific functions during odontogenesis. We screened that the expression level of circRNA_ID:4:55530561-55530959 was 6.7 times more than that in the control group, which indicates its close relationship to odontoblast differentiation. According to its sequence, we confirmed that this circRNA was derived from the third exon of *Klf4*. As a newly discovered circRNA absent from circBase,^1^ we term it circKLF4. Concerning the important function of its parental gene, *Klf4*, during odontoblastic differentiation (Lin et al., 2013; Tao et al., 2019), we supposed that circKLF4 might be closely related to the odontoblast differentiation.

In this study, we investigated the role circKLF4 plays during the odontoblastic differentiation of mDPCs and the underlying molecular mechanism during this process.

## Materials and Methods

The entire study satisfied the requirements of the Ethics Committee of the School of Stomotology, Wuhan University (protocol 00266935).

### Cell/Tissue Isolation and Culture

The primary mDPCs were separated from Kunming mice (China) at embryonic day (E) 18.5, digested with 3 mg/mL trypsin and cultured in Dulbecco modified Eagle medium (DMEM; Hyclone) supplemented with 10% fetal bovine serum (FBS; Gibco) and 1% penicillin/streptomycin (Hyclone). mDPCs were seeded in a 12-well plate at an initial density of 1 × 10^5^ cells/well. The culture medium was changed every 2–3 days as previously described (Lin et al., 2013). The dental pulp tissues were isolated from mandibular molar teeth of six Kunming mice (China) at postnatal day (PN) 21.

### Odontoblastic Differentiation of mDPCs

For odontoblastic differentiation induction, mDPCs were incubated with 1 mL of DMEM supplemented with 10% FBS, 1% penicillin/streptomycin, 50 μg/mL ascorbic acid (Sigma-Aldrich), 10 mmol/L β-glycerophosphate (Sigma-Aldrich) and 10 nmol/L dexamethasone (Sigma-Aldrich) after the cells became confluent.

### *In situ* Hybridization

Kunming mice were sacrificed for sample collection at PN 1. Tissues for *in situ* hybridizations were dissected and fixed in 4% paraformaldehyde. Samples were followed by paraffin embedding and sectioning. Digoxin-labeled specific targeting the mouse circKLF4 probe was prepared and the *in situ* hybridization procedures were performed as described earlier (Yuan et al., 2009). Briefly, hybridization was performed at 65–68°C overnight in a solution containing 50% formamide, 0.5 mM EDTA (pH 8), formamide (50%), 20 × SSC (pH 4.5), yeast RNA (50 mg/mL), heparin (10 mg/mL), 0.1% Tween 20, CHAPS (10%) and circKLF4 probe. After hybridization, add anti–digoxygenin (DIG)-alkaline phosphatase (AP) antibody at 4°C overnight. Wash the slides with freshly made NTMT buffer and then incubate them in BM purple and develop color in dark, humid environment at 4°C for several days. Stop reaction with washing in PBS and dehydrate slides, then mount with mounting medium and capture images.

### circKLF4 and Dicer Knockdown, Plasmid Construction

The sequences of circKLF4 siRNA oligonucleotides (circKLF4-si) (GenePharma) are 5′-UGGGGGAAGUCGCUUGUUGTT-3′ (sense) and 5′-CAACAAGCGACUUCCCCCATT-3′ (anti-sense). Silencer select negative control siRNA (GenePharma) was used as the control. *Dicer* siRNA oligonucleotides were purchased from Thermo.

The circKLF4 sequence was cloned into the pcDNA 3.1 (+) circRNA Mini Vector (*P*-vector) to construct its overexpression plasmid (P-circKLF4). The mutated circKLF4 plasmids (P-circKLF4-Mut-1895 and P-circKLF4-Mut-5046) were created using the Fast Mutagenesis Kit (Vazyme).

After 3–4 days’ culture of mDPCs, siRNA, miRNA inhibitor (30 pmol, GenePharma) and wild type or mutant circKLF4 plasmid were co-transfected into cells with Lipofectamine 2,000 (Invitrogen).

### Real-Time RT-PCR

Total RNAs were isolated using the HP Total RNA Kit (Omega Bio-tech) according to the manufacturer’s protocol and was transcribed into cDNA using the Revert Aid First Strand cDNA Synthesis Kit (Thermo Scientific). The cDNA samples were then amplified using FastStart Universal SYBR Green Master (Rox) with primers (Table 1). Total microRNA was isolated using miRNeasy mini Kit (QIAGEN) and was transcribed into cDNA using the miSript Reverse Transcription Kit (QIAGEN). The cDNA samples were amplified using miSript SYBR Green PCR Kit (QIAGEN) with primers (Table 1). The cDNA samples were assayed using the CFX Connect Real Time PCR Detection System (Bio-Rad). The relative gene expression levels of circRNA and mRNA were normalized to GAPDH primers, and levels of microRNA were normalized to U6 primers using the 2^–Δ^ ^Δ^ ^Ct^ method. The gene expression ratio was determined from three independent experiments.

**TABLE 1:** Oligonucleotide primer sequences utilized in RT-qPCR.

Gene	Forward primer	Reverse primer
*Gapdh*	F: TGTGTCCGTCGTGGATCTGA	R: TTGCTGTTGAAGTCGCAGGAG
*Alp*	F: TCATTCCCACGTTTTCACATTC	R: GTTGTTGTGAGCGTAATCTACC
*Dmp1*	F: CTGTCATTCTCCTTGTGTT CCTTTG	R: CAAATCACCCGTCCTC TCTTCA
*Dspp*	F: ATCATCAGCCAGTCAGAA GCAT	R: TGCCTTTGTTGGGACC TTCA
*Klf4*	F: GGGAAGTCGCTTCATGTG AGAG	R: GCGGGAAGGGAGAAGA CACT
*circKLF4*	F: TCGCTTCATGTTGAAGGGGG	R: CCTGCAGCTTCAGCTATCCG
*Dicer*	F: AGCTCCGGCCAACACCTTTA	R:GTGTACCGCTATGAAATCATTGA
*Endoglin*	F: CACAGCTGCACTCTGGTACA	R: GGAGGCTTGGGATACTCACG

### Western Blot

Cells were lysed in protein lysis buffer (Beyotime). The whole cell lysis products were analyzed with SDS-PAGE and then transferred to PVDF membrane (Millipore). The following primary antibodies were used: anti-KLF4 polyclonal antibody (ab106629, Abcam), anti-DMP1 polyclonal antibody (ab103203, Abcam), anti-DSP polyclonal antibody (NBP191612, NOVUS), and anti-β-ACTIN monoclonal antibody (660091Ig, Proteintech). After incubation with the corresponding antibodies, the membrane was washed 3 times for 5 min each with TBST. We used ImageJ software for further densitometric analysis. Bound primary antibodies were detected by incubating for 1 h with horseradish peroxidase conjugated goat anti-mouse or anti-rabbit IgG (Biofly, China) for analysis. The membrane was washed and developed by a chemiluminescence assay (GE Healthcare).

### Statistical Analysis

All experiments were independently repeated at least 3 times and the results are presented as mean ± standard deviation (SD). Differences between two groups were analyzed with a 2-tailed *t*-test with *p-*values less than 0.05 indicated significant. Data analysis were performed by GraphPad Prism 5.0 software (San Diego, CA, United States).

## Results

### circKLF4 Promotes the Odontoblastic Differentiation of mDPCs

**FIGURE 1:**
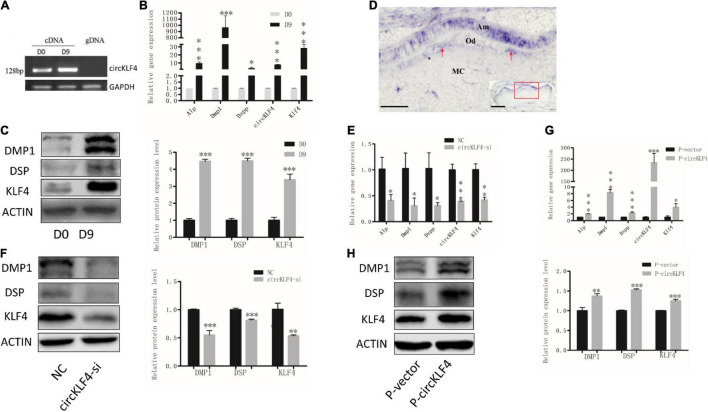
circKLF4 promotes the odontoblastic differentiation of mDPCs. **(A)** The existence of circKLF4 was validated by gel electrophoresis using the products of quantitative real-time PCR. **(B)** mRNA levels of *Alp, Dmp1, Dspp*, circKLF4, and *Klf4* by real-time reverse transcription polymerase chain reaction in the mDPCs cultured in differentiation medium. **(C)** Western blot and quantification of the relative protein levels of DMP1, DSP, KLF4 cultured in differentiation medium. **(D)** *In situ* hybridization of circKLF4 in PN1 mouse molar. **(E)** mRNA levels of *Alp, Dmp1, Dspp*, circKLF4, and *Klf4* after transfection with circKLF4 siRNA (circKLF4-si) compared with the control group (NC). **(F)** DMP1, DSP, KLF4 levels and quantified density after circKLF4 was knocked down. **(G)** mRNA levels of *Alp, Dmp1, Dspp, Klf4*, and circKLF4 after transfected with circKLF4 overexpression plasmid (*P*-circKLF4) compared with the empty vector (*P*-vector). **(H)** The protein levels of DMP1, DSP, and KLF4 after overexpression of circKLF4. Right panel shows the quantified data. Significant difference vs. day 0, *P < 0.05; ^**^P < 0.01; ^***^P < 0.001. Scale bars = 100 μm for **(D)**.

In order to determine whether circKLF4 is associated with odontoblast differentiation, the expression level of circKLF4 was detected in mDPCs cells during odontoblastic differentiation. Specific primers covering the head-to-tail splicing site of circKLF4 were designed. The specificity of the primers was confirmed by PCR, with cDNA and genomic DNA (gDNA) as the template. As expected, PCR products were only amplified in cDNA but not in gDNA (Figure 1A), which suggests that the primers are specific for circKLF4 but not for linear KLF4. qPCR results showed that circKLF4 was significantly upregulated on day 9 in primary mDPCs (Figure 1B). Furthermore, the mRNA and protein expression levels of odontoblastic-related genes (*Alp*, *Dmp1*, *Dspp*, and *Klf4*) were significantly upregulated on day 9, which confirmed the odontoblastic differentiation of the cells (Figures 1B,C). The expression level of circKLF4 in dental pulp cells of erupted molar of 3 weeks old was also investigated and we found that the expression levels of both circKLF4 and linear *Klf4* in mouse dental pulp cells were significantly up-regulated compared with that in the mDPCs (Supplementary Figure 1A). Then the expression pattern of circKLF4 in the mouse lower molar at PN1 was detected *in vivo*, using *in situ* hybridization. As showed in Figure 1D, circKLF4 displayed at a low level in the papilla but was expressed intensely and specifically in odontoblasts and ameloblasts.

To determine the biological function of circKLF4 in odontoblastic differentiation, a small interfering RNA specifically targeting the back-splicing junction site of circKLF4 (circKLF4-si) was designed to knock down circKLF4. circKLF4 was substantially decreased at 48 h after transfection with circKLF4-si (Figure 1E). The expressions of the odontoblast marker genes were also substantially down-regulated both in mRNA and protein levels (Figures 1E,F). Furthermore, these odontoblast marker genes were upregulated both in mRNA and protein levels with overexpression of circKLF4 (Figures 1G,H).

### circKLF4 Increases the Linear *Klf4* Expression in a microRNA-Dependent Manner

**FIGURE 2:**
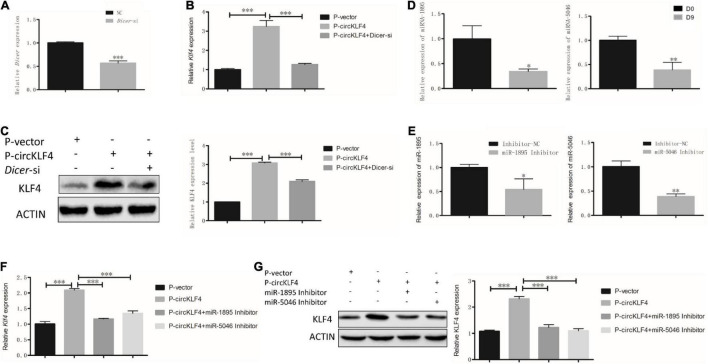
circKLF4 up-regulates liner *Klf4* expression to promote Odontoblastic Differentiation in a microRNA-dependent manner. **(A)** mRNA level of *Dicer* was significantly down-regulated after *Dicer*-si was transfected. **(B,C)** Compared with overexpression of circKLF4 alone, mRNA and protein levels of *Klf4* were significantly down-regulated after co-transfected with *Dicer*-si. **(D,E)** miR-1895 and miR-5046 was analyzed by RT-PCR after 9 days’ differentiation induction or using the inhibitors. **(F,G)** mRNA and protein levels of *Klf4* were significantly down-regulated after co-transfection of miR-1895/miR-5046 inhibitor with *P*-circKLF4. *P < 0.05; ^**^P < 0.01; ^***^P < 0.001.

Since KLF4 operates as a switch-triggering odontoblast differentiation (Feng et al., 2017), Figure 1 showed circKLF4 was able to up-regulate linear KLF4 expression in the gain- and loss- of circKLF4 experiments, which indicates circKLF4 promotes odontoblastic differentiation of mDPCS *via* upregulation of linear KLF4. Then the mechanism of how circKLF4 regulates linear KLF4 was explored. As circRNAs can regulate gene expression by sponging microRNAs, to determine whether circKLF4 can increase the liner *Klf4* expression by sponging certain microRNAs, we knocked down Dicer, an enzyme required for cleavage of precursor miRNAs, to decrease mature miRNAs (Song and Rossi, 2017). qRT-PCR results showed that *Dicer* was significantly knocked down with *Dicer* siRNA (*Dicer-*si) transfection (Figure 2A). Overexpression of circKLF4 increased the expression of *Klf4* in mRNA and protein levels, but co-transfection with *Dicer* siRNA partially abolished this effect (Figures 2B,C), suggesting that the regulation of linear *Klf4* by circKLF4 is in a microRNAs-dependent manner.

**FIGURE 3:**
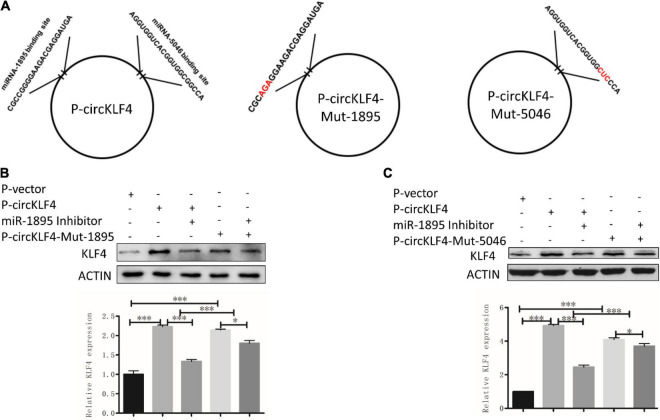
circKLF4 promotes liner Klf4 expression by competitively binding miR-1895 and miR-5046. **(A)** The left scheme demonstrated the predicted binding sites of miR-1895 and miR-5046 in *P*-circKLF4. The right two diagrams showed the characteristics of mutant-type P-circKLF4 (*P*-circKLF4-Mut-1895 and *P*-circKLF4-Mut-5046). **(B,C)** Western blot and qPCR results showed the expression of KLF4 after transfected with or without P-circKLF4, miR-1895 or miR-5046 inhibitor, *P*-circKLF4-Mut-1895 or *P*-circKLF4-Mut-5046. *P < 0.05; ^***^P < 0.001.

To screen the certain microRNAs binding to circKLF4, 10 predicted miRNAs were acquired by mirBase^2^ and listed in Supplementary Table 1. miRNA-1895 and miRNA-5046 were found to be among the top 2 miRNAs of the list. The binding sequences of miR-1895 and miR-5046 on linear *Klf4* were shown in Supplementary Figure 1. Given that these sequences are shared by both circKLF4 and linear *Klf4* in miR-1895 and miR-5046 (Figure 3A and Supplementary Figure 1B), we hypothesized that circKLF4 might promote liner *Klf4* expression by sponging miR-1895 or miR-5046.

The RT-PCR results showed that both miRNA-1895 and miRNA-5046 were down-regulated at day 9 (Figure 2D). To further elucidate the function of miR-1895 and miR-5046 during the regulation of linear KLF4 by circKLF4, the inhibitors of miR-1895 and miR-5046 were used and knock-down efficiency was confirmed by qPCR (Figure 2E). Furthermore, we transfected the miR-1895 inhibitor and/or miR-5046 inhibitor into mDPCs with or without overexpression of circKLF4. Overexpression of circKLF4 increased the mRNA and protein levels of linear *Klf4*, but co-transfection with miR-1895 inhibitor or miR-5046 inhibitor could abolished this effect (Figures 2F,G). These results indicated that circRNA circKLF4 upregulates linear KLF4 *via* miR-1895 and miR-5046.

### circKLF4 Promotes Liner *Klf4* Level by Competitively Binding miR-1895 and miR-5046

To further confirm whether circKLF4 regulates linear *Klf4* level by competitively binding miR-1895 and miR-5046, we mutated the binding site of miR-1895 and miR-5046 in circKLF4 overexpression plasmid (Figure 3A) and transfected the mutants into mDPCs with/without co-transfection of miR-1895/miR-5046 inhibitor. The western blot results showed that transfection of either wild type circKLF4 expression plasmid or circKLF4-mut-1895 plasmid was able to upregulate KLF4 expression (Figure 3B, lane 2 and 4). Co-transfection of miR-1895 inhibitor was able to effectively abolish the upregulation effect of wild type circKLF4 expression plasmid to linear KLF4 expression (Figure 3B, compared lane 3 and 2), but was not so effective to abolish the upregulation effect of circKLF4-mut-1895 plasmid to linear KLF4 expression (Figure 3B, compared lane 5 and 4). The similar results were found in the transfection of circKLF4-mut-5046 and miR-5046 inhibitor (Figure 3C). These results indicated that circKLF4 regulates linear KLF4 expression by competitively binding miR-1895 and miR-5046.

### circKLF4 Also Promotes Endoglin Expression *via* Sponging miR-1895 and miR-5046

**FIGURE 4:**
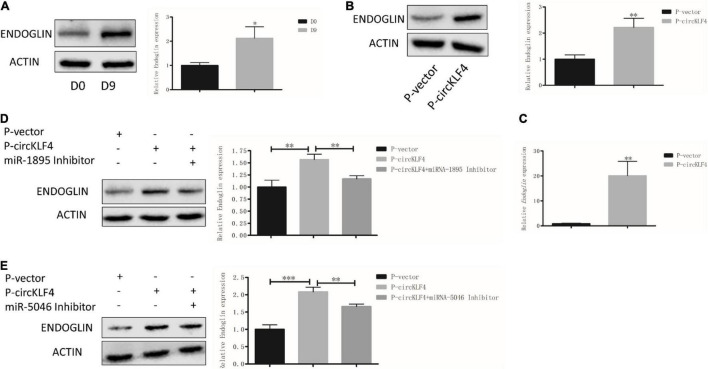
circKLF4 also promotes Endoglin expression *via* sponging miR-1895 and miR-5046. **(A)** Protein level of ENDOGLIN was significantly up-regulated after 9 days’ induction. **(B,C)** The protein and mRNA levels of ENDOGLIN were significantly up-regulated after transfected with *P*-circKLF4 compared with the *P*-vector group. **(D,E)** Western blot results showed the expression level of ENDOGLIN after co-transfected of miR-1895 or miR-5046 inhibitor with *P*-circKLF4. *P < 0.05; ^**^P < 0.01; ^***^P < 0.001.

Since circKLF4 could competitively bind miR-1895 and miR-5046, furthermore, miRNA could regulate multiple genes’ expression (Panda, 2018). Thus, we wondered if it is possible that circKLF4 can regulate odontoblastic differentiation by regulating other genes except for *Klf4* through miR-1895 and miR-5046. By querying the miRNA target gene prediction and functional analysis database TargetScan,^3^ we found that both miRNA-1895 and miRNA-5046 have binding sites with *Endoglin* by binding its 3′ untranslated region (3′UTR) (Supplementary Figure 1C). Endoglin has been identified to participate in odontoblast differentiation in the previous studies. In our *in vitro* odontoblastic differentiation experiments, western blot results showed that ENDOGLIN was significantly increased on day 9 (Figure 4A). Overexpression of circKLF4 upregulated both the protein and mRNA levels of *Endoglin* (Figures 4B,C). However, co-transfection of miR-1895/miR-5046 inhibitor abolished this effect (Figures 4D,E). These results showed, besides KLF4, circKLF4 also promotes Endoglin expression *via* miR-1895 and miR-5046 to induce odontoblastic differentiation of mDPCs.

## Discussion

**FIGURE 5:**
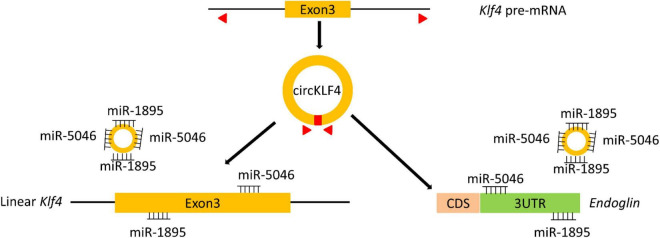
A scheme indicates that circKLF4 upregulates *Klf4* and *Endoglin* to promote odontoblastic differentiation of mDPCs *via* sponging miRNA-1895 and miRNA-5046.

The study of odontoblastic differentiation is essential to understand the process of tooth development and to achieve tooth regeneration in the future. Emerging evidence has revealed that circRNAs participate in odontoblast differentiation (Li and Jiang, 2019). Here, we reported and elucidated the potential role of a circRNA termed circKLF4 during odontoblast differentiation. We demonstrated that circKLF4 modulated the expression of KLF4 and ENDOGLIN to promote odontoblastic differentiation by sponging miRNA-5046 and miRNA-1895 mechanistically (Figure 5).

As we know, the studies about circRNAs in odontoblast differentiation are still limited (Li and Jiang, 2019). A recent study demonstrated that 1,314 and 1,780 circRNAs were upregulated and downregulated in human dental pulp cells during odontogenic differentiation (Li and Jiang, 2019). In our preliminary circRNA sequencing data, we also found the differential expression of circRNAs in dental papilla cells after differentiation. In this study, we identified that circKLF4 was upregulated in differentiated odontoblasts with 9 days’ induction of differentiation. Our *in situ* hybridization result showed that circKLF4 expression was intense in the odontoblasts and ameloblasts, but was at a low level in the mesenchyme cells. Similarly, as the parental gene of circKLF4, KLF4 was also detectable in polarizing odontoblasts and ameloblasts in the first molar at PN1 in the previous investigation (Chen et al., 2009). The similar expression patterns of circKLF4 and linear KLF4 indicated the possibility of regulation between circKLF4 and KLF4. Besides, KLF4 has been identified to promote odontoblast differentiation both *in vitro* and *in vivo* (Tao et al., 2019), which leads us to explore the role of circKLF4 during odontoblastic differentiation. In the present investigation, gain- and loss-of-circKLF4 confirmed that circKLF4 could up-regulate KLF4 expression and promote odontoblast differentiation.

Accumulating investigations have implied that circRNAs act as miRNA sponges to regulate gene expression. MicroRNA are predicted to regulate protein-coding genes (Berezikov et al., 2005). The miRNA genes are transcribed into primary miRNA (pri-miRNA) to generate pre-miRNA, which is then processed by Dicer to produce mature miRNA (Siomi and Siomi, 2010). As Dicer could inhibit the formation of miRNAs (Song and Rossi, 2017), with transfection of *Dicer* siRNA we revealed that known-down of Dicer abolished the upregulation of KLF4 by circKLF4, suggesting that circKLF4 modulated KLF4 expression in a miRNAs-dependent manner. The targeted microRNAs binding to circKLF4 were predicted by mirBase, miRNA-1895 and miRNA-5046 were found to be the top 2 miRNAs. Accumulating evidence indicates that miRNAs participate in odontoblast differentiation (Sun et al., 2015) and circRNAs are shown to regulate gene expression by inhibiting miRNA activity (Panda, 2018). Our previous study showed that miR-1895 inhibited the odontoblastic differentiation (Zhang and Yang, 2021), suggesting that circKLF4 might be a miR-1895 antagonist with a miR-1895-binding capacity. To our knowledge, the function of miR-5046 during odontoblast differentiation has not been studied. However, RT-PCR data demonstrated that miR-1895 and miR-5046 were both downregulated in the differentiated mDPCs. Several other assays, including gain- and loss-of-circKLF4, use of microRNAs inhibitor and mutation of the binding sites of the microRNAs in circKLF4 overexpression plasmid were also performed, which indicated that circKLF4 regulated KLF4 expression *via* sponging miR-1895 and miR-5046.

As miRNA could target the 3′ UTRs of specific mRNA targets (Pillai, 2005) and regulate multiple genes’ stability and/or translation (Panda, 2018). By querying the miRNA target gene prediction and functional analysis database TargetScan (see text footnote 3), we found that both miR-1895 and miR-5046 have binding sites with the 3′ UTR region of the gene *Endoglin*. So it is predicted that circKLF4 could also regulate *Endoglin* expression *via* miR-1895 and miR-5046. Endoglin, also called CD105, which is ubiquitously expressed in mesenchymal stem cells. Besides, the Endoglin positive selection has been proposed for the isolation of DPSC (dental pulp stem cells) and Endoglin expression was detected in odontoblasts of human (Huang et al., 2010). A previous study showed that, *Endoglin* was found to be upregulated in DPSCs during the induction of DPSCs into dentin-secreting odontoblast-like cells (Liu et al., 2007) and involved in osteogenic differentiation of periodontal ligament cells (Ishibashi et al., 2010). Consistent with our study, we also identified that *Endoglin* was significantly increased with odontoblastic differentiation in mDPCs and overexpression of circKLF4 increased the expression levels of *Endoglin*. Besides, co-transfection with miR-1895 or miR-5046 could inhibited the up-regulation of ENDOGLIN by overexpression of circKLF4, indicating the potential role of miRNA during the regulation of ENDOGLIN by circKLF4.

To summarize, we identified that circKLF4 was up-regulated in differentiated mDPCs and promoted odontoblast differentiation through up-regulation of KLF4 and ENDOGLIN by sponging miR-1895 and miR-5046.

## Data Availability Statement

The original contributions presented in the study are included in the article/Supplementary Material, further inquiries can be directed to the corresponding author/s.

## Ethics Statement

The animal study was reviewed and approved by the Ethics Committee of the School of Stomotology, Wuhan University.

## Author Contributions

YZ made contributions to data acquisition, analysis, draft, and critical revision of the manuscript. HZ contributed to advice, analysis, and discussions. GHY conceived, analyzed the experiments, and critically revised the manuscript. GBY contributed to conception, design, analysis, and critically revised the manuscript. All authors approved the manuscript and agreed to be accountable for the work.

## Conflict of Interest

The authors declare that the research was conducted in the absence of any commercial or financial relationships that could be construed as a potential conflict of interest.

## Publisher’s Note

All claims expressed in this article are solely those of the authors and do not necessarily represent those of their affiliated organizations, or those of the publisher, the editors and the reviewers. Any product that may be evaluated in this article, or claim that may be made by its manufacturer, is not guaranteed or endorsed by the publisher.

## Footnotes

1
http://www.circbase.org/

2
http://www.mirbase.org/

3
http://www.targetscan.org/

## Supplementary Material

Supplementary Figure 1 **(A)** mRNA levels of *circKLF4 and Klf4* were significantly up-regulated in mouse dental pulp of PN21, compared with those in mouse dental papilla cells of PN1. **(B,C)** A scheme shows the potential binding sites between miR-1895 or miR-5046 and the *Klf4* or *Endoglin*.

## References

[B1] Ashwal-Fluss R. Meyer M. Pamudurti N. R. Ivanov A. Bartok O. Hanan M. (2014). circRNA biogenesis competes with pre-mRNA splicing. *Mol. Cell* 56 55–66. 25242144 10.1016/j.molcel.2014.08.019

[B2] Berezikov E. Guryev V. van de Belt J. Wienholds E. Plasterk R. H. Cuppen E. (2005). Phylogenetic shadowing and computational identification of human microRNA genes. *Cell* 120 21–24. 10.1016/j.cell.2004.12.031 15652478

[B3] Capel B. Swain A. Nicolis S. Hacker A. Walter M. Koopman P. (1993). Circular transcripts of the testis-determining gene Sry in adult mouse testis. *Cell* 73 1019–1030. 10.1016/0092-8674(93)90279-y 7684656

[B4] Chen Z. Couble M. L. Mouterfi N. Magloire H. Chen Z. Bleicher F. (2009). Spatial and temporal expression of KLF4 and KLF5 during murine tooth development. *Arch. Oral Biol.* 54 403–411. 10.1016/j.archoralbio.2009.02.003 19268913

[B5] Dassule H. R. Lewis P. Bei M. Maas R. McMahon A. P. (2000). Sonic hedgehog regulates growth and morphogenesis of the tooth. *Development* 127 4775–4785. 11044393 10.1242/dev.127.22.4775

[B6] Feng J. Jing J. Li J. Zhao H. Punj V. Zhang T. (2017). BMP signaling orchestrates a transcriptional network to control the fate of mesenchymal stem cells in mice. *Development* 144 2560–2569. 10.1242/dev.150136 28576771 PMC5536932

[B7] Greene J. Baird A. M. Brady L. Lim M. Gray S. G. McDermott R. (2017). Circular RNAs: biogenesis, function and role in human diseases. *Front. Mol. Biosci*. 4:38. 10.3389/fmolb.2017.00038 28634583 PMC5459888

[B8] Gu X. Li M. Jin Y. Liu D. Wei F. (2017). Identification and integrated analysis of differentially expressed lncRNAs and circRNAs reveal the potential ceRNA networks during PDLSC osteogenic differentiation. *BMC Genet.* 18:100. 10.1186/s12863-017-0569-4 29197342 PMC5712120

[B9] Huang G. T. Yamaza T. Shea L. D. Djouad F. Kuhn N. Z. Tuan R. S. (2010). Stem/progenitor cell-mediated de novo regeneration of dental pulp with newly deposited continuous layer of dentin in an in vivo model. *Tissue Eng. Part A* 16 605–615. 10.1089/ten.TEA.2009.0518 19737072 PMC2813150

[B10] Ishibashi O. Ikegame M. Takizawa F. Yoshizawa T. Moksed M. A. Iizawa F. (2010). Endoglin is involved in BMP-2-induced osteogenic differentiation of periodontal ligament cells through a pathway independent of Smad-1/5/8 phosphorylation. *J. Cell. Physiol.* 222 465–473. 10.1002/jcp.21968 19918795

[B11] Li C. Jiang H. (2019). Altered expression of circular RNA in human dental pulp cells during odontogenic differentiation. *Mol. Med. Rep.* 20 871–878. 10.3892/mmr.2019.10359 31173232 PMC6625184

[B12] Lin H. Liu H. Sun Q. Yuan G. Zhang L. Chen Z. (2013). KLF4 promoted odontoblastic differentiation of mouse dental papilla cells via regulation of DMP1. *J. Cell. Physiol*. 228, 2076–2085. 10.1002/jcp.24377 23558921

[B13] Liu J. Jin T. Chang S. Ritchie H. H. Smith A. J. Clarkson B. H. (2007). Matrix and TGF-beta-related gene expression during human dental pulp stem cell (DPSC) mineralization. *In Vitro Cell. Dev. Biol. Anim*. 43 120–128. 10.1007/s11626-007-9022-8 17516126

[B14] Memczak S. Jens M. Elefsinioti A. Torti F. Krueger J. Rybak A. (2013). Circular RNAs are a large class of animal RNAs with regulatory potency. *Nature* 495 333–338. 10.1038/nature11928 23446348

[B15] Panda A. C. (2018). Circular RNAs Act as miRNA Sponges. *Adv. Exp. Med. Biol.* 1087 67–79. 10.1007/978-981-13-1426-1_6 30259358

[B16] Pasman Z. Been M. D. Garcia-Blanco M. A. (1996). Exon circularization in mammalian nuclear extracts. *RNA* 2 603–610. 8718689 PMC1369399

[B17] Pillai R. S. (2005). MicroRNA function: multiple mechanisms for a tiny RNA? *RNA* 11 1753–1761. 10.1261/rna.2248605 16314451 PMC1370863

[B18] Qian D. Y. Yan G. B. Bai B. Chen Y. Zhang S. J. Yao Y. C. (2017). Differential circRNA expression profiles during the BMP2-induced osteogenic differentiation of MC3T3-E1 cells. *Biomed. Pharmacother.* 90 492–499. 10.1016/j.biopha.2017.03.051 28395271

[B19] Salzman J. Chen R. E. Olsen M. N. Wang P. L. Brown P. O. (2013). Cell-type specific features of circular RNA expression. *PLoS Genet.* 9:e1003777. 10.1371/journal.pgen.1003777 24039610 PMC3764148

[B20] Salzman J. Gawad C. Wang P. L. Lacayo N. Brown P. O. (2012). Circular RNAs are the predominant transcript isoform from hundreds of human genes in diverse cell types. *PLoS One* 7:e30733. 10.1371/journal.pone.0030733 22319583 PMC3270023

[B21] Siomi H. Siomi M. C. (2010). Posttranscriptional regulation of microRNA biogenesis in animals. *Mol. Cell* 38 323–332. 10.1016/j.molcel.2010.03.013 20471939

[B22] Song M. S. Rossi J. J. (2017). Molecular mechanisms of Dicer: endonuclease and enzymatic activity. *Biochem. J.* 474 1603–1618. 10.1042/BCJ20160759 28473628 PMC5415849

[B23] Sun J. Li B. Shu C. Ma Q. Wang J. (2020). Functions and clinical significance of circular RNAs in glioma. *Mol. Cancer* 19:34. 10.1186/s12943-019-1121-0 32061256 PMC7023692

[B24] Sun Q. Liu H. Chen Z. (2015). The fine tuning role of microRNA-RNA interaction in odontoblast differentiation and disease. *Oral Dis.* 21 142–148. 24654877 10.1111/odi.12237

[B25] Szabo L. Morey R. Palpant N. J. Wang P. L. Afari N. Jiang C. (2015). Statistically based splicing detection reveals neural enrichment and tissue-specific induction of circular RNA during human fetal development. *Genome Biol*. 16:126. 10.1186/s13059-015-0690-5 26076956 PMC4506483

[B26] Tao H. Lin H. Sun Z. Pei F. Zhang J. Chen S. (2019). Klf4 promotes dentinogenesis and odontoblastic differentiation *via* modulation of TGF-beta signaling pathway and interaction with histone acetylation. *J. Bone Miner. Res.* 34 1502–1516. 10.1002/jbmr.3716 31112333 PMC8895434

[B27] Thesleff I. Partanen A. M. Kuusela P. Lehtonen E. (1987). Dental papilla cells synthesize but do not deposit fibronectin in culture. *J. Dent. Res.* 66 1107–1115. 10.1177/00220345870660060401 3305632

[B28] Yuan G. Wang Y. Gluhak-Heinrich J. Yang G. Chen L. Li T. (2009). Tissue-specific expression of dentin sialophosphoprotein (DSPP) and its polymorphisms in mouse tissues. *Cell Biol. Int.* 33 816–829. 10.1016/j.cellbi.2009.05.001 19450697 PMC2725224

[B29] Zhang H. Yang G. (2021). Regulation of odontoblastic differentiation by miR-1895 in mouse dental papilla cells. *J. Oral Sci. Res.* 37 28–32. 10.13701/j.cnki.kqyxyj.2021.01.007

[B30] Zhang Y. D. Chen Z. Song Y. Q. Liu C. Chen Y. P. (2005). Making a tooth: growth factors, transcription factors, and stem cells. *Cell Res.* 15 301–316. 15916718 10.1038/sj.cr.7290299

