# Supplementary figures and images for: circKLF4 Upregulates Klf4 and Endoglin to Promote Odontoblastic Differentiation of Mouse Dental Papilla Cells via Sponging miRNA-1895 and miRNA-5046
Source: Front Physiol. 2022 Feb 9;12:760223. doi: 10.3389/fphys.2021.760223 (PMC8865004; doi:10.3389/fphys.2021.760223)

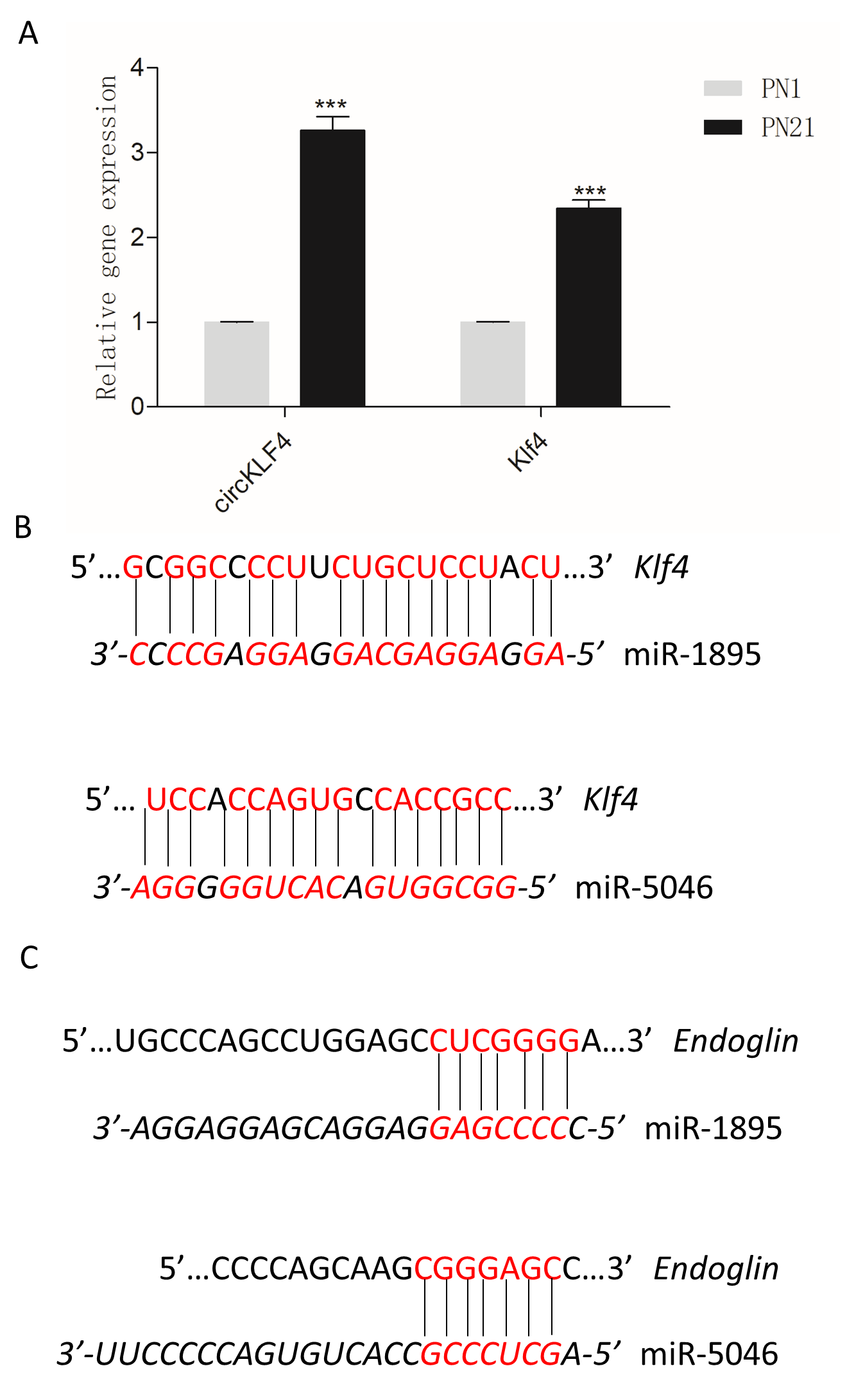

Supplement: Supplementary Figure 1 — (A) mRNA levels of circKLF4 and Klf4 were significantly up-regulated in mouse dental pulp of PN21, compared with those in mouse dental papilla cells of PN1. (B,C) A scheme shows the potential binding sites between miR-1895 or miR-5046 and the Klf4 or Endoglin. [file Image_1.TIF]
